# Formulation of New Baking (+)-Catechin Based Leavening Agents: Effects on Rheology, Sensory and Antioxidant Features during Muffin Preparation

**DOI:** 10.3390/foods9111569

**Published:** 2020-10-29

**Authors:** Gabriele Carullo, Francesca Scarpelli, Emilia Lucia Belsito, Paolino Caputo, Cesare Oliviero Rossi, Antonio Mincione, Antonella Leggio, Alessandra Crispini, Donatella Restuccia, Umile Gianfranco Spizzirri, Francesca Aiello

**Affiliations:** 1 Department of Biotechnology, Chemistry and Pharmacy, Department of Excellence 2018–2022, University of Siena, Via Aldo Moro 2, 53100 Siena, Italy; gabriele.carullo@unisi.it; 2 Department of Pharmacy, Health and Nutritional Sciences, Department of Excellence 2018–2022, University of Calabria, Edificio Polifunzionale, 87036 Rende, Italy; emilialucia.belsito@unical.it (E.L.B.); antonella.leggio@unical.it (A.L.); umile_gianfranco.spizzirri@unical.it (U.G.S.); francesca.aiello@unical.it (F.A.); 3 Department of Chemistry and Chemical Technologies, University of Calabria, 87036 Arcavacata di Rende-Cosenza, Italy; francesca.scarpelli@unical.it (F.S.); paolino.caputo@unical.it (P.C.); cesare.oliviero@unical.it (C.O.R.); alessandra.crispini@unical.it (A.C.); 4 AGRARIA Department, Mediterranean University of Reggio Calabria, Loc. Feo di Vito, 89122 Reggio Calabria, Italy; amincione@unirc.it

**Keywords:** muffin, baking powder, formulation and characterization, rheology, antioxidant performances, sensory properties, (+)-catechin

## Abstract

The aim of this investigation was to prepare two solid mixtures containing a soluble polymorph of (+)-catechin and mucic (MUC) or tartaric (TAR) acids as new leavening agents. The solid mixtures were based on a polymorph of (+)-catechin, characterized through Powder X-ray Diffraction (PXRD) analysis and assayed in *in vitro* antioxidant and solubility assays. The dough samples were studied by dynamic rheological tests, while muffins were studied through Headspace Solid-Phase Microextraction (HS-SPME)/Gas Chromatography-Mass Spectrometry (GC-MS) analysis to identify volatile compounds, *in vitro* tests to evaluate antioxidant properties, and sensory analyses. TAR powder showed a solubility in water almost one order of magnitude increased with respect to commercial (+)-catechin (40.0 against 4.6 mg mL^−1^) and increased antioxidant performances. In particular, TAR showed total phenolic content (TPC) and total antioxidant capacity (TAC) values of 0.0298 ± 0.021 and 0.0081 ± 0.0009 meq CT/g, while MUC showed better results in terms of 2,2-diphenyl-1-picrylhydrazyl) acid (DPPH) and 2,2′-azino-bis (3-ethylbenzothiazoline-6-sulphonic acid (ABTS), 0.518 ± 0.015 and 0.112 ± 0.010 mg/mL, respectively. MS analysis identified different compounds derived from the lipid oxidation process. Muffins obtained using both powders showed interesting outcomes regarding dough process and appreciable appearance/olfactory/taste/texture profiles. Muffins obtained from TAR-based mixture showed also a total phenolic content of 0.00175 meq CT/g muffin, and almost two times improved TAC and scavenger activity against DPPH radical. The formulated powders could be used as suitable health-promoting ingredients in the food industry.

## 1. Introduction

Baked products are widely consumed worldwide. Among them, muffins rank third in breakfast products as they are highly appreciated for their texture and taste [1]. Basic ingredients for muffin production are wheat flour, sugar, milk, fat, and egg added with a leavening agent to drive the baking process. The latter is probably the key factor to obtain a valuable product, producing heat and mass transfer supported by temperature rising and gas production (i.e., CO_2_). The high temperature also induces the formation of a dry surface crust as well as many chemical and physical reactions converting the cake batter into an expanded volume crumb with positive organoleptic features [2].

Many parameters affect the baking process, including ingredient proportion and handling, time, temperature, heating systems, and delivery, as well as the leavening additive employed. In particular, the latter aspect shows dramatic effects on sensory, rheological, and textural features of the final product [3]. Among leavening agents, baking powders are widely used in muffin preparation. Commercial formulations are generally a mixture of inorganic compounds (baking soda and acid salts) that react in presence of moisture and heat to release CO_2_. Starch is usually added for stability and moisture control. Baking powders differ from one another in relation to diverse acidic constituents. Most of the common acid components are phosphate salts, showing the drawback of negatively affecting the flavor of the final product [4]. Moreover, although phosphates are normal constituents in the body and are regular components of the diet, a re-evaluation of the safety of phosphates-based food additives (E 338–341, E 343, and E 450–452) has been recently conducted by the European Food Safety Authority (EFSA), leading to a final scientific opinion [5]. The EFSA Panel on Food Additives and Flavorings, proposed a group Acceptable Daily Intake (ADI) for phosphates of 40 mg kg^−1^ bw per day expressed as phosphorus (P), suitable for healthy individuals, although not enforceable to humans with moderate to severe reduction in renal function (about 10% of the population). Moreover, based on analytical data, the Panel underlined that P dietary exposure exceeded the proposed ADI for infants, toddlers, and children at the mean level and for infants, toddlers, children, and adolescents at the 95th percentile; the same conclusion was drawn for food supplements. It follows that, considering the wide diffusion of baked products, replacing phosphates with different leavening ingredients could limit P dietary exposure for better meeting the EFSA proposed dietary intake.

For a further improving of muffins’ nutritional value, fortification with polyphenols could be also considered a valuable approach as these compounds have been widely recognized to elicit many health-promoting effects [6]. Flavonoids addition was exploited in the food sector [7,8,9,10,11], also showing in baked products an important antiglycation activity [12,13]. However, flavan-3-ols suffer poor oral bioavailability, implying too high plasma concentrations needed to bring out an assessable impact on health [14]. Several strategies can be adopted to enhance solubility and hence bioavailability of flavonoids [15]; for example, it has been demonstrated that the use of an absorption enhancer, such as apple pectin, increases the intestinal absorption of quercetin, a poorly soluble flavonoid [16]. Moreover, co-crystallization was proved to be effective and some studies reported the application of co-crystals of polyphenols for food and beverage fortification [17,18]. In addition, phospholipid complexation of flavanols can have a positive impact on their oral absorption [19]. The pharmaceutical industry has often taken advantage of polymorphism as a method to overcome bioavailability issues of poorly soluble active ingredients, since it is generally recognized that different polymorphic forms of a chemical compound can display different physicochemical properties; in particular, water-solubility [20]. Moreover, the conversion of an active ingredient from a polymorphic form to another can be gained through simple experimental methods, such as solvent-crystallization and grinding.

Flavonoids can exist in more crystalline forms [21,22], including solvates and hydrates [23,24,25]; however, to the best of our knowledge, flavonoids polymorphism has not yet been applied in the field of food and beverage fortification.

On this basis, in this study the (+)-catechin (CT) polymorphism has been exploited in order to prepare two multifunctional solid mixtures, containing a soluble form of (+)-catechin (CT) and mucic acid (MA) or tartaric acid (TA) employed as baking ingredients in muffin preparation.

Catechins are phytochemical compounds found in high concentrations in a variety of plant-based foods and beverages. More precisely, catechin is the 3,3′,4′,5,7-pentahydroxyflavan with two steric forms. The (+)-catechin was selected due to its exceptional positive effects related to human health. Among these, catechins have function as an anti-angiogenic antitumor agent, modulator of tumour cell response to chemotherapy, and anti-inflammatory action in bowel disease by direct and indirect inhibition of oxidative stress [26,27,28]. Galactaric acid, also known as mucic acid (MA), is a symmetrical six carbon diacid, used to produce a gum alternative to arabic gum. Currently, MA is commercially produced by oxidation of galactose with nitric acid or from D-galacturonic acid (D-galUA) by electrolytic oxidation. A useful alternative source of D-galUA is pectin, an abundant component in fruit peels that represents an available and inexpensive source for production of MA [29]. Tartaric acid is an important food additive naturally occurring in plants like grapes, apricots, apples, bananas, avocados, and tamarinds. Its salt potassium bitartrate, commonly known as cream of tartar, develops naturally in the process of fermentation. It is usually mixed with sodium bicarbonate to form a baking powder used as a leavening agent in food preparation. TA can exist in different isomeric forms, but only L (+)-tartaric acid and its salts are authorized as food additives labelled as E334. In this context, to valorise catechin health effects and safe use, as well as the sustainable recovery of both acids from agro-food wastes (grape pomace for L (+)-tartaric acid and citrus pectin for MA), we decided to formulate these two new baking powders. In this new baking powder, the acidic counterpart necessary to guarantee the leavening effect, exerted most commonly by calcium acid phosphate, sodium aluminumsulfate were substituted by Tartaric acid and Mucic acid, mixed with alkali (sodium bicarbonate is known commonly as baking soda) and a polymorph of catechin. By adding water to this mixture, a chemical reaction is achieved, producing carbon dioxide, which is trapped in tiny air pockets in the dough or batter.

To the best of our knowledge, this was the first attempt to verify the effectiveness of an alternative leavening agent able, at the same time, to carry on a product fortification. After X-ray characterisation of the multi-functional solid mixtures, their antioxidant features and their effects on batter rheology were investigated, while, to highlight the effect on the quality of the final product, antioxidant activity, flavor compounds and sensory characteristics were evaluated on muffins samples.

## 2. Materials and Methods

### 2.1. Chemicals

(+)-Catechin, ascorbic acid, gallic acid, tartaric acid, mucic acid, sodium hydrogen carbonate, starch, Folin–Ciocalteu reagent, sodium carbonate, sulphuric acid, sodium phosphate, ammonium molybdate, 2,2-diphenyl-1-picrylhydrazyl) acid (DPPH), 2,2′-azino-bis (3-ethylbenzothiazoline-6-sulphonic acid (ABTS), and formic acid were purchased from Sigma-Aldrich (Milford, MA, USA).

n-Hexane, methanol, water, and ethanol 96% were purchased from Merck (Darmstad, Germany) and VWR International (Milan, Italy) and, unless specified otherwise, were of analytical grade or higher.

### 2.2. Synthesis of Baking Powders Through Slurry Process

In a round-bottom flask, CT (500 mg, 1.72 mmol) was slurred with TA (129 mg, 0.86 mmol) or MA (181 mg, 0.86 mmol) in 5 mL of ethanol, for 24 h at room temperature, in a dark atmosphere. The round-bottom flasks were left in the hood undisturbed, and, after two days, two solids were obtained, named CT_TA and CT_MA. To obtain the correspondent baking powders (labelled M_BP and T_BP, respectively) about 800 mg of CT_TA and CT_MA were mixed with 800 mg of sodium hydrogen carbonate and 2.4 g of starch, in order to obtain 4.0 g each of the two baking powders.

### 2.3. Powder X-ray Diffraction (PXRD) ofCT, CT_TA and CT_MA

The PXRD analysis was performed using a Bruker D2-Phaser operating at 30 kV and 10 mA, using Cu-Kα radiation (λ = 1.5418 Å) and equipped with a Lynxeye detector. The PXRD patterns were acquires over an angular range of 5–45° 2θ, with a step size of 0.01°. The obtained diffraction profiles were analyzed with DIFFRAC.EVA diffraction software (version 3.1, Bruker AXS 2010-2013, Billerica, MA, USA).

### 2.4. Antioxidant Performances of MUC, TAR and COM

#### 2.4.1. Total Phenolic Content

The total phenolic content (TPC) in the synthetic (M_BP and T_BP) and commercial baking powders (C_BP) was detected according to the Folin–Ciocalteu colorimetric method [30,31]. Briefly, a volume of each sample (6.0 mL) and the Folin–Ciocalteu reagent (1.0 mL) were mixed and, after 3 min, 3.0 mL of Na_2_CO_3_ (5.0% *w*/*v*) was added. Positive control and blank solutions was also prepared by substituting the sample with the same volume of 0.1% (*w*/*v*) ascorbic acid and hydro alcoholic solution (50:50 *v*/*v*), respectively. The absorbance value of each solution was recorded after 2 h under stirring at 760 nm by a Jasco V-530 UV/VIS Spectrometer (Jasco, Tokyo, Japan). The TPC was expressed as milliequivalent of CT per g of baking. A calibration curve was recorded by six gallic acid standard solutions. Each solution (6.0 mL) was added to the Folin–Ciocalteu reagent to raise the following final concentrations: 5.0, 10.0, 20.0, 40.0, 60.0, and 100.0 × 10^−6^ mol L^−1^. UV-Vis analyses were performed after 120 min of incubation under stirring and correlation coefficient, slope and intercept of the regression equation obtained by the method of least squares were calculated.

#### 2.4.2. Determination of Total Antioxidant Capacity

A literature protocol, with few changes was employed to determine total antioxidant capacity (TAC) of each baking agent [32]. Briefly, an aliquot of hydro-alcoholic solution (50:50 *v*/*v*) of each sample (0.3 mL) (M_BP, T_BP and C_BP) was mixed to 1.2 mL of reagent solution (0.6 mol L^−1^ H_2_SO_4_, 28.0 mol L^−1^ Na_3_PO_4_, and 4.0 mol L^−1^ (NH_4_)_2_MoO_4_) at 95 °C, and after 150 min the absorbance was recorded at 695 nm. Ascorbic acid 0.1% (*w*/*v*) was used as positive control. The total antioxidant activity of each baking powder was expressed as milliequivalent of CT equivalent per g of sample. A calibration curve was recorded by six catechin standard solutions. Each solution (0.3 mL) was added to 1.2 mL of reagent solution in order to raise the following concentrations: 5.0, 10.0, 20.0, 40.0, 60.0, and 100.0 × 10^−6^ mol L^−1^. UV-Vis analyses were performed after 150 min of incubation, and correlation coefficient, slope, and intercept of the regression equation obtained by the method of least squares were calculated.

#### 2.4.3. Determination of Scavenging Activity on DPPH Radicals

Baking agents (M_BP, T_BP and C_BP) scavenger activities towards DPPH radical were evaluated following a literature protocol with some modifications [33]. Briefly, equivolumetric hydroalcoholic solution of each sample (1.0 mL), equivolumetric hydroalcoholic mixture (4.0 mL) and 200 μmol L^−1^ ethanol DPPH solution (5.0 mL) were mixed in a volumetric flask at 25 °C and, after 24 h, the absorbance was recorded at 517 nm. Ascorbic acid 0.1% (*w*/*v*) was used as positive control. Scavenging properties of the tested matrices were expressed as IC_50_ (the concentration of sample that causes a 50% decrease in the starting DPPH concentration).

#### 2.4.4. Determination of Scavenging Effect on the ABTS Radical Cation

Baking agents (M_BP, T_BP and C_BP) scavenging properties were evaluated in aqueous media towards ABTS radical [34]. In a general procedure, 500 μL of hydroalcoholic solution (50:50 *v*/*v*) on each baking powder were added to 2.0 mL of the ABTS^+^ radical solution (7.0 mmol L^−1^ properly diluted to raise approximately an absorbance of 0.70 at 734 nm). The mixture was incubated at 37 °C for 5 min and the absorbance was recorded at 734 nm. A positive control based on an ascorbic acid 0.1% (*w*/*v*) solution was employed. The absorbance decrease of the ABTS radical cation provided the antioxidant activity of the baking powders, expressed as IC_50_ (the concentration of sample that causes a 50% decrease in the starting ABTS concentration).

### 2.5. Preparation of Muffins

Ingredients: 1 egg (M measure, AIA), partially skimmed milk UHT(Parmalat) (100 g), white sugar Eridania(100 g), 00 wheat flour Barilla(100 g), sunflower oil Carapelli (100 g) and 4 g of M_BP, T_BP or 8 g of C_BP Lievito PaneAngeli.

**Figure 1:**
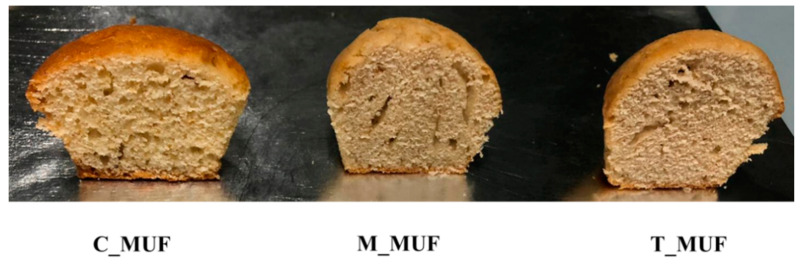
Representative samples of muffins obtained using the new leavening agents (M_MUF, T_MUF and commercial one C_MUF).C_MUF = Muffin prepared by C_BP; M_MUF = Muffin prepared by M_BP; T_MUF = Muffin prepared by T_BP.

Method: the egg was manually brought with a whisk into a glass bowl, sugar was added and the mixture was beaten until a clear and frothy consistency is reached. Then, the oil was added to the mixture. In another bowl, baking and wheat flour were mixed, sifted, and gradually this mixture was added to the dough. Finally, milk was added. Into twelve compartments of a sanitized aluminum muffin pan (each chamber: 5.0 cm top diameters, 5.0 cm bottom, 4.0 cm height) about 40.0 ± 0.5 g of muffin butter were placed. They were cooked in a preheated domestic oven REX ^®^Electrolux under static mode of the oven at 180 °C for 25–30 min to obtain the corresponding muffins labelled as M_MUF, T_MUF, and C_MUF (Figure 1).

### 2.6. Rheological Measurements

Rheological measurements were done using a shear strain-controlled rheometer RFS III (Rheometrics, Piscataway, NJ, USA) equipped with a parallel plate geometry (gap 2.0 ± 0.1 mm, diameter 50 mm for the samples analyzed within the temperature range 15–25 °C) and a Peltier system (±0.1 °C) for temperature regulation. Transient experiments yielded steady flow experiment results, according to step-rate tests where the viscosity was measured at different shear rate values in the set time. The small amplitude dynamic tests (frequency sweep tests) provided information on the linear viscoelastic behaviour of materials through the determination of the complex shear modulus (Equation (1))

G*(ω) = G′(ω) + **i**G″ (ω) (1)

where the complex modulus (G*), represents a quantitative measure of material stiffness or resistance to deformation, G′(ω) represents a measure of the reversible, elastic energy, while G″(ω) stands for the irreversible viscous dissipation of the mechanical energy and i is the imaginary unit of the complex number [35,36]. The applied strain amplitude for the viscoelastic measurements was reduced until the linear response regime was reached. This analysis was done by carrying out strain sweep tests in all investigated temperature ranges [37].

### 2.7. Antioxidant Performances of Muffin

Antioxidant properties of muffins were evaluated by performing polyphenol extraction procedure reported in literature with some modification [38]. Five grams of each muffin (M_MUF, T_MUF and C_MUF) were accurately weighed and defatted with *n*-hexane (150 mL, 20 min, 70 °C). The n-hexane fraction was decanted and the defatted sample was extracted in 40 mL of a mixture (70% methanol, 29.7% water, and 0.3% formic acid), at 70 °C for 45 min under stirring, and then filtered, evaporated, and finally dried under vacuum until constant weight.

TPC and antioxidant performances of the muffins’ extracts were performed following the procedures previously reported. Specifically, TPC and TAC were expressed as milligram of CT per gram of muffin, while scavenger activities in organic and aqueous environments were evaluated as IC_50_ values.

### 2.8. Extraction of Volatile Compounds from Muffins by Headspace Solid-Phase Microextraction (HS-SPME)

For HS-SPME analysis, a 10 mL vial containing a fixed amount (2.5 g) of each type of freshly baked muffin was sealed with a PTFE/silicone septum and heated in a 30 °C thermostatic water bath for 30 min. Once thermal equilibrium had been attained, a 50/30 μm DVB/CAR/PDMS (divinylbenzene/carboxen/polydimethylsiloxane) SPME fiber was used to sample the headspace above the solid matrix. DVB/CAR/PDMS coating is a polymeric multiphase containing both absorbing (PDMS) and adsorbing (DVB and CAR) phases able to trap polar and non-polar compounds. Headspace sampling was conducted for 3 h at 30 °C. After this time, the analytes on the fiber were thermally desorbed for 10 min at 250 °C in the injector of the Gas Chromatography -Mass Spectrometry (GC-MS) system, then they were separated and identified. Prior to use, the fibers were thermally conditioned in the GC injector according to manufacturer’s instructions. The thermal equilibrium time was optimized monitoring from 15 to 60 min the extraction efficiency, using a constant extraction time of 15 min. After 30 min a relatively constant response was observed up to 60 min. Therefore, 30 min was chosen as thermal equilibrium time. Headspace sampling and related GC/MS analysis were performed in triplicate for each sample type.

### 2.9. GC-MS Analyses

The analyses were carried out on an Agilent GC/MS system consisting of a 6890 N Network gas chromatograph and a 5973Network Mass Selective Detector (5973N MSD) operating in 70 eV electron impact ionization mode. Mass spectra were acquired in full scan mode in the range of 40–650 *m*/*z*.

The GC was equipped with a 30 m HP-5MS capillary column (0.25 mm i.d., 0.25 µm film thickness) and helium was used as the carrier gas at a rate of 1 mL/min. The injector was set at 250 °C and was used in splitless mode with a splitless time of 1 min. The oven temperature program was set at an initial temperature of 40 °C (hold time 10 min), then temperature ramp of 4 °C/min up to 210 °C and ramp of 30 °C/min to reach a final temperature of 220 °C (final hold time 5 min). The compounds were tentatively identified based on their Electron Ionization (EI) mass spectra using the NIST08 database. The identities of some detected volatile compounds were also confirmed by comparing EI mass spectra and chromatographic retention times with those of available standards. Blank experiments, including blank of the fiber and blank of the empty vial, were accomplished after each analysis in order to rule out the presence of any parasitic signals. The relative peak area percentage of each compound is achieved by the ratio between the peak area for that compound and the sum of the peak areas of all the compounds identified in the chromatogram.

### 2.10. Sensory Analysis

Muffins (M_MUF, T_MUF, and C_MUF) were evaluated by descriptive sensory analysis by a trained panel of 8 adult judges (5 males and 3 females, aged between 23 and 65 years, recruited among departmental faculty staff). Samples belonging to each of the three different muffin types were served in a standard sensory booth in random order unknown to the judges at room temperature, with data averaged over three replicates. Assessors were trained according to ISO 8586:2012 [39] guidelines for the selection, training and monitoring of expert sensory assessors. Additional training was given with reference products to address specific taste and appearance descriptors. Panellists cleansed with mineral water and non-salted crackers between samples. Training for muffin sensory attributes was given with commercially available muffins one day before actual test. Judges rated samples on a 10-point structured scale from 0 to 9 for appearance, smell, taste and texture descriptors (Table 1), with a score 0 indicating the absence of the attribute and 9 an extremely high attribute value.

### 2.11. Statistical Analysis

GC-MS and antioxidant results were reported as mean ± standard deviation (SD) (three replicates). Moreover, antioxidant data underwent one-way analysis of variance (ANOVA) using Prism GraphPad Prism version 4.0 for Windows, GraphPad Software (San Diego, CA, USA). After that, the Tukey’s test was performed to compare all means. Differences between the means were statistically significant with *p* < 0.05.

Regarding sensory analysis, mean results are reported in the form of spider plot graph. Descriptive data were also submitted to Principal Component Analysis (PCA). PCA analysis on sensory data was performed on all descriptor data, by using the NIPALS algorithm and with Varimax data rotation. (The Unscrambler, version 10.4, CAMO, Norway).

## 3. Results and Discussion

### 3.1. Preparation and PXRD Analysis

The solids (CT_MA and CT_TA) were prepared through the slurry process in ethanol, to obtain multifunctional solid mixtures, in which the properties of the single components remain unchanged. In particular, MA and TA were chosen for their baking activity, whereas catechin was selected for its well-known antioxidant properties [40]. Therefore, in analogy with the well-known studies of drug–excipients compatibility [41,42] physical and chemical interactions between the single components within the CT_MA and CT_TA solid mixtures are undesired since they could affect the individual properties.

**Figure 2:**
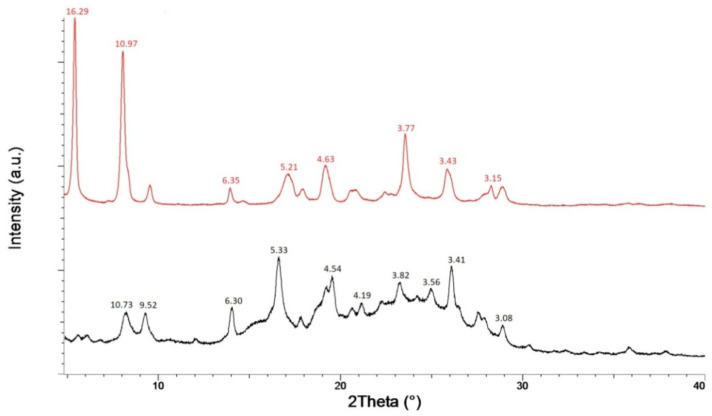
PXRD patterns of CT before (black line) and after (red line) slurry process. Position of the main peaks reported in Å. CT = (+)-catechin.

The commercially available CT, according to its characteristic PXRD profile (Figure 2, black line), consists in the β-monohydrate form, which, unfortunately, displays poor water solubility [23] and therefore poor bioavailability. However, it is well known that water recrystallization of CT in its β-monohydrate polymorph can induce a phase transition into the tetrahydrate form [24]; moreover, additional catechin polymorphs, with different water solubility, have been observed, including several hydrates [25], an anhydrous form and methanol solvate form. On this basis, before preparing the baking powders formulation, the flavonoid β-monohydrate polymorph was slurred individually in ethanol, with the aim of investigating if a polymorphic transition occurs and, eventually, which solvate and/or hydrated form is generated. The eventual formation of the ethanol solvate would represent a great improvement in the CT bioavailability, since, according to the general rules of polymorphs solubility [26] the ethanol solvate should be more soluble than the monohydrate phase.

The resulting microcrystalline powder was analyzed through Powder X-Ray Diffraction, in order to determine the eventual CT structural modification induced by the slurry process. Indeed, the PXRD profile of CT after slurry in ethanol is found different from that of the β-monohydrate form, indicating the occurred solid state phase transition (Figure 2, red line).

**Figure 3:**
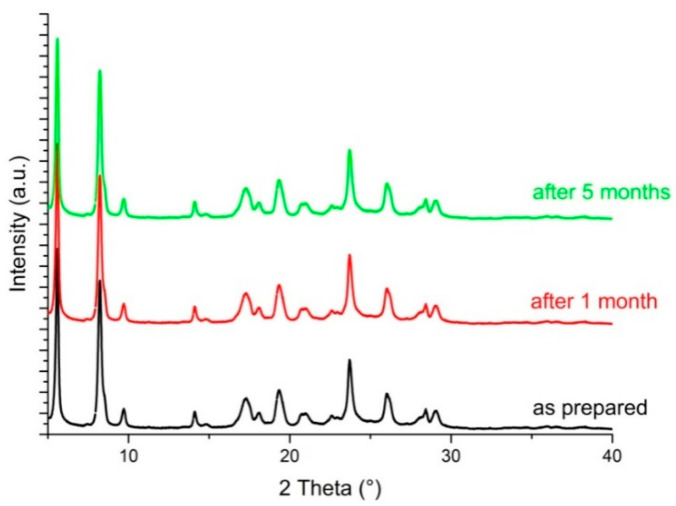
PXRD pattern of (+)-catechin tetrahydrate recorded as prepared (black line), after 1 month (red line) and after 5 months (green line).

However, the new PXRD pattern corresponds to the tetrahydrate form of catechin [23], excluding the formation of a new ethanol solvate form. It is important to highlight that the tetrahydrate form of catechin displays higher water solubility than the β-monohydrate. Since, to the best of our knowledge, water solubility quantitative measurements of the catechin tetrahydrate form have never been reported, the water solubility of the obtained resulting powder was measured and compared with that of the β-monohydrate form; in particular the solubility values in water are 4.6 mg mL^−1^ for catechin β-monohydrate and 8.0 mg mL^−1^ for catechin tetrahydrate. Moreover, although generally a soluble polymorph is less stable than the insoluble one, converting to the thermodynamically more stable and less soluble form [27] in this case, the catechin tetrahydrate polymorph is very stable, as demonstrated by the absence of any change in its PXRD pattern recorded over time (Figure 3).

**Figure 4:**
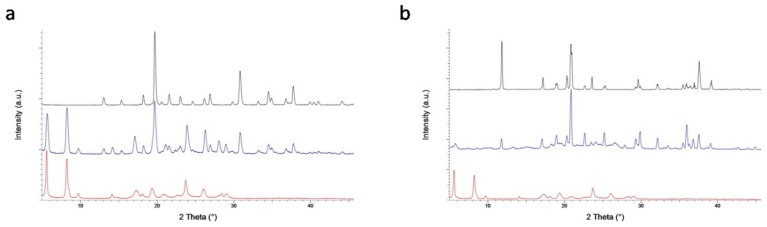
PXRD patterns of: (**a**) CT tetrahydrate (red line), the solid mixture CT_MA (blue line) and MA (black line); (**b**) CT tetrahydrate (red line), the solid mixture CT_TA (blue line) and TA (black line). CT = (+)-Catechin; CT_MA = (+)-catechin_mucic acid adduct; MA = Mucic acid. CT_TA = (+)-catechin_tartaric acid adduct; TA = Tartaric acid.

**Figure 5:**
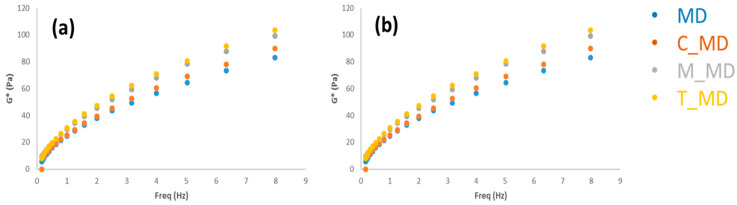
Frequency Sweep Test muffin dough (G *). (**a**): 15 °C, (**b**): 25 °C. MD = muffin dough without baking; T_MD = muffin dough with T_BP; M_MD = muffin dough with M_BP; C_MD = muffin dough with C_BP.

In order to verify the absence and/or presence of intermolecular interactions between the flavonoid CT and the two organic acids (MA and TA), the PXRD profiles of the solid mixtures, CT_MA and CT_TA, were acquired and compared to those of their relative components (Figure 2 and Figure 3). As expected, the slurry process induces the flavonoid solid phase conversion, thus, the PXRD patterns of the sample CT_MA and CT_TA may be regarded as the overlap of CT tetrahydrate and MA and TA diffractograms (Figure 4 and Figure 5, respectively). The formation of CT tetrahydrate during the slurry process determines an increase of the water-solubility of CT_MA and CT_TA with respect to monohydrate form of the flavonoid; in particular, the solubility values in water are 6.0 and 40.0 mg mL^−1^ for CT_MA and CT_TA, respectively.

Thus, the presence of the distinctive diffraction peaks of the individual component in both samples (CT_MA and TA_CT) diffractograms, and the absence of new reflections allow us to exclude the formation of any intermolecular interactions, with eventual formation of new supramolecular species, between the flavonoid and the two organic acids. Hence, since the individual components remain unchanged after the treatment, it is reasonable to assume that they may retain their individual properties within the solid state mixtures.

### 3.2. Antioxidant Performances of Baking Additives

Antioxidant properties of the phenolic compounds makes these molecules very interesting due to their ability to prevent different human diseases related to the oxidative stress [43]. Moreover, in recent years the consumers’ attention was particularly focused to the knowledge of the antioxidant performances of the consumed foods [44]. To this regard, the baking agents prepared by CT_MA and CT_TA can be a useful tool to make bakery products with remarkable antioxidant properties. Total phenolic content and antioxidant properties of M_BP and T_BP were investigated and the results reported in Table 2. Additionally, C_BP was employed as control.

The results display the baking powders showing interesting antioxidant properties as a consequence of the introduction of molecules having polyphenol structure. On the contrary, C_BP do not show any activity. Specifically, all the assays underlined as antioxidant performances of M_BP were statistically better than T_BP (*p* < 0.05). This trend was particularly evident by observing the IC_50_ value related to the scavenger activity in the organic environment, against DPPH radical, with a value almost five times lower for M_BP with respect to T_BP.

### 3.3. Rheological Characterization

Dynamic rheological tests (frequency sweep experiments), at 15 and 25 °C, respectively, were carried out to study the dough samples. Macroscopically, one usually observes a liquid structure for the samples. Strain-sweep experiments that are not reported here revealed that all samples have a nonlinear response at a considerably higher strain. The dynamic moduli for all samples lose linearity at ca. 100% strain at a frequency of 1 Hz.

The mechanical spectrum reflects the texture structure of the system. In Figure 5, the rheological spectra were reported for all samples. It is worthy to note differences in G* (Figure 5) where all values are higher than those of muffin dough without baking (WY). It is also important to note that MUC and TAR samples show similar rheological behaviour. Their frequency profiles are higher than those of other ones. These results indicate that the synthesized baking affects the structures of the system and improve the mechanical properties of the doughs.

The variation of viscosity versus time at different shear rates for samples was measured (Figure 5). It was observed that different samples have different rheological behaviors. A thinning behavior is shown, where the viscosity is dependent on shear rates. Higher shear rates reduce the viscosity such as in a shear-thinning fluid, also known as a pseudo plastic system. The shear thinning behavior appears increasingly pronounced when tartaric is used. The pseudo plastic fluid and higher viscosity can be attributed to higher structure-forming interactions [45,46].

**Figure 6:**
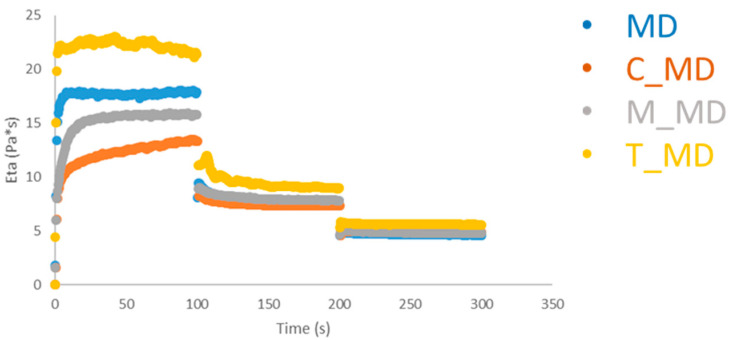
Step Rate muffin dough at 15 °C. MD = muffin dough without baking; T_MD = muffin dough with T_BP; M_MD = muffin dough with M_BP; C_MD = muffin dough with C_BP.

In Figure 6, the frequency sweep tests of baked muffins achieved employing the synthetic baking (M_MUF and T_MUF) and commercial one (C_MUF) are reported. It is worthy to note the effects of different baking on the rheological properties of the final products. The trends are almost flat in frequency (the structure is formed by large network thus a high coordination number); additionally, the higher values as a function of the frequency observed for T_MUF and M_MUF can be related to the higher “interaction strength” between the rheological units (stronger links).

This means that the synthetic baking ingredients (M_MUF and T_MUF) form more structured samples [47].

**Figure 7:**
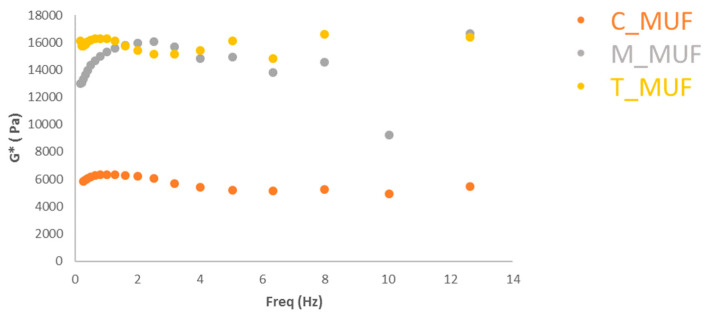
Frequency Sweep Test at 25 °C muffin. C_MUF = Muffin prepared by C_BP; M_MUF = Muffin prepared by M_BP; T_MUF = Muffin prepared by T_BP.

Similar trends are observable between them, while they are different in comparison with the C_MUF sample (Figure 7).

### 3.4. Antioxidant Performances of Muffins

The use of antioxidant baking powders could represent an innovative tool to achieve antioxidant food products [7]. To this regard, muffins underwent an extraction procedure in order to attain extracts rich in polyphenols [38]. The extracts were investigated to determine the antioxidant performances of the final product and the results are reported in Table 3.

The data clearly showed that the employment of functional baking ingredients led to a product with antioxidant properties, with respect to the control C_MUF that did not display any activity. Specifically, the analysis of TPC allows us to note that the highest value was recorded for T_MUF (0.00175 meq CT/g muffin), while the best performing compound was M_BP. This different trend certainly underlines the role organic acid in the prevention of the antioxidant feature loss during the preparation of the muffins, i.e., catechin oxidation, isomerization, and degradation. In particular, the catechinisomerization could easily take place under the stated processing conditions, strongly depending on temperature and pH [48]. Higher values of pH and temperature decrease the stability of the catechin [49].

The data regarding TPC values was confirmed in the analyses of the antioxidant performances of the products. TAC and scavenger activities, both in organic and aqueous environments, highlighted T_MUF as the best performing product concerning the antioxidant properties. In detail, TAC and scavenger activity against DPPH radical was almost two times improved in T_MUF, while in an aqueous environment against ABTS radical, the IC_50_ value recorded for M_MUF appears almost one order of magnitude higher than T_MUF.

A comparison of the recorded data with other enriched muffins is very difficult because usually active molecules were added to the dough. On the contrary, our approach is quite different and allowed us to impart antioxidant features to the final product by using a baking agent with antioxidant peculiarities. Recently, muffins enriched with different amounts of a by-product of chia oil extraction (2.5–10% *w*/*w*) or pomegranate peel supplementation (5–10% *w*/*w*) showing antioxidant features that displayed TPC value and antioxidant performances in the same order of magnitude of the products proposed in this research [50,51].

### 3.5. Analysis of Volatile Compounds in Muffins

The approach used for the determination of the volatile fraction in muffins is based on the headspace analysis by solid-phase microextraction (HS-SPME) technique [52,53] combined with gas chromatography/mass spectrometry (GC/MS), analytical technique chosen in order to separate and identify the different extracted volatile compounds [54,55].

The volatile compounds of muffin samples (M_MUF, T_MUF, and C_MUF), were extracted from the solid matrix by DVB/CAR/PDMS fiber and then analyzed by GC/MS.

**Figure 8:**
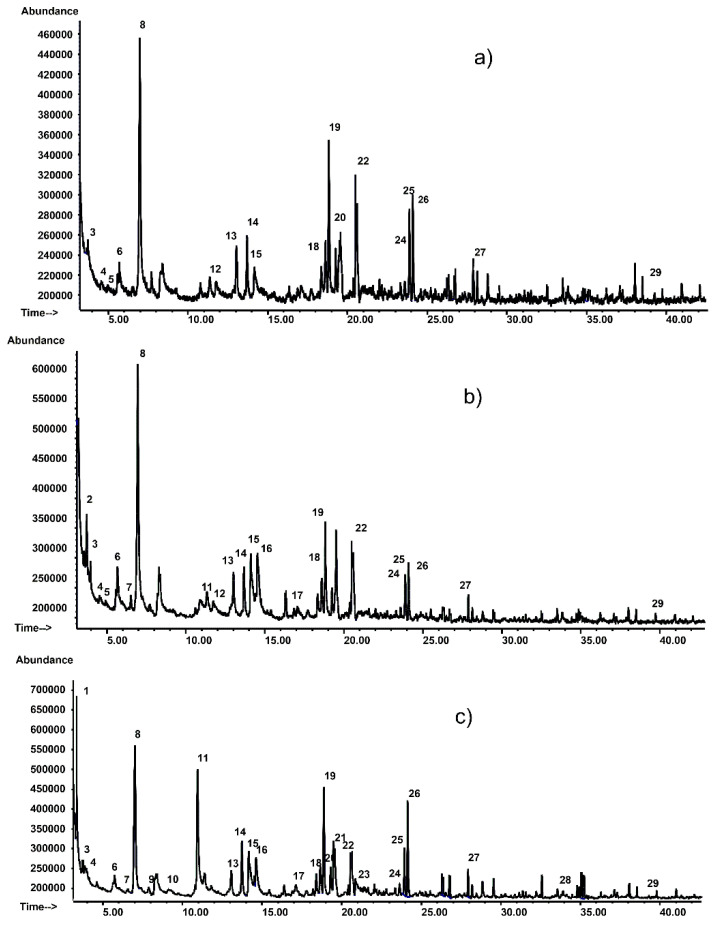
HS-SPME/GC–MS (EI) analysis of: (**a**) muffin sample M_MUF; (**b**)muffin sample T_MUF; (**c**) muffin sample C_MUF. M_MUF = Muffin prepared by M_BP; T_MUF = Muffin prepared by T_BP; C_MUF = Muffin prepared by C_BP. Numbers 1-29 indicate the identified compounds reported in Table 4.

In Figure 8 are reported the GC profiles obtained analysing the muffin samples (M_MUF, T_MUF, C_MUF).

In the GC profiles (Figure 8) of the analyzed samples, about 29 volatile compounds, belonging to different chemical classes, were tentatively identified by matching the corresponding mass spectra to those of the mass spectral library or by comparing retention times and mass spectra with those of available standards (Table 4). The relative peak area percentage for each compound was defined as the ratio between the peak area for that compound and the sum of all identified peaks areas in that sample.

The obtained aromatic profiles are due to several factors, mainly related to the lipid oxidation and thermal degradation of sugars and free amino acids through caramelization and Maillard reactions during cooking. They are characterized by the presence of aldehydes, hydrocarbons, ketones, alcohols, furans, pyrazines, and sulphur-containing compounds.

Concerning aldehydes, the presence of 3-methylbutanal, 2-methylbutanal, hexanal, nonanal, heptanal, 2-furancarboxaldehyde, octanal, and benzaldehyde was observed (Table 4). 3-methylbutanal (t_R_ = 3.72 min., analyte 3, Table 4), detected in all muffin samples and responsible of malty odour, is an aldehyde deriving from oxidative deamination and decarboxylation of leucine in the presence of carbonyl compounds (Strecker degradation), while 2-methylbutanal (Sample T_MUF) is originated from isoleucine. In Table 4, it can be observed that hexanal, a typical compound of linoleic and arachidonic acids oxidation, is the major aldehyde component in all analyzed muffin samples, followed by nonanal, heptanal, and octanal, the latter of which is detected only in C_MUF, which are formed by oxidation of oleic acid [56].

Benzaldehyde, detected in samples T_MUF and C_MUF, which can be considered a Strecker aldehyde, originated from the reaction of the parent α-amino acid (phenylglycine) with α-dicarbonyl compounds from lipid decomposition [56].

In the group of ketones—in addition to identifying in all muffin samples 2-heptanone and 2-nonanone mainly originating from lipid oxidation—was detected, exclusively in C_MUF muffin, the compound 1-hydroxy-2-propanone formed through the Strecker degradation of proline. This molecule is a precursor of α-aminoketones that on auto-condensation and subsequent oxidation and condensation with fatty aldehydes and ketones generate pyrazine derivatives [57]. Alkylpyrazines can be also originated from the Maillard reactions of free amino acids and reducing sugars.

Pyrazine (t_R_ = 4.56 min., analyte 4, Table 4) and 2,5-dimethylpyrazine (t_R_ = 14.15 min., analyte 15, Table 1), were observed in all three muffin types (Table 4).

In the group of heterocyclic compounds, besides identification of pyrazine compounds, pyridine (t_R_ = 4.91 min., analyte 5, Table 4) and 2,5-dibutylthiophene (t_R_ = 39.74 min., analyte 29, Table 4) were detected. Heterocyclic compounds containing sulphur and nitrogen atoms are mainly formed through the interaction of Maillard reaction intermediates with lipid decomposition products such as 2,4-decadienal and hexanal.

Substituted-furans such as 2-furancarboxaldehyde, 2-furanmethanol, 2(5H)-furanone and 2-pentylfuran were also detected (Table 4). These compounds are formed through the complex processes of Maillard reaction and sugar thermal degradation during baking [58]. 2-pentylfuran is the only furan derivative detected in all muffin samples (t_R_ = 18.84 min., analyte 19, Table 4). The formation of 2-pentylfuran could be attributed both to Maillard reaction and lipid oxidation [59].

Hydrocarbon compounds like toluene, 1,3-dimethylbenzene, decane, undecane and dodecane detected in all muffin samples (Table 4), could come from the oxidative decomposition of lipids and are often associated with grassy and fatty flavors [60].

Among the volatile components that contribute to the aroma of all analyzed muffin samples we also found 1-octen-3-ol (t_R_ = 18.36, analyte 18, Table 4), a compound with a strong mushroom note that is associated with the enzymatic degradation of linoleic acid [61]. Other detected alcohols are 1-pentanol (sample T_MUF and C_MUF) and 2-ethyl-1-hexanol (sample C_MUF).

Some volatile molecules like 1-hydroxy-2-propanone, methyl pyrazine, 2-furancarboxaldehyde, 2-ethyl-1-hexanol, octanal, and tetradecane (Table 4) were detected exclusively in the extract of C_MUF.

In the GC profile of the control (C_MUF) is observed the presence of a higher number of volatile components compared to T_MUF and M_MUF muffins.

The obtained results show that volatile compounds that derive from lipid oxidation and thermal degradation of sugars and free amino acids affect the flavor of these products.

### 3.6. Muffin Sensory Analysis

**Figure 9:**
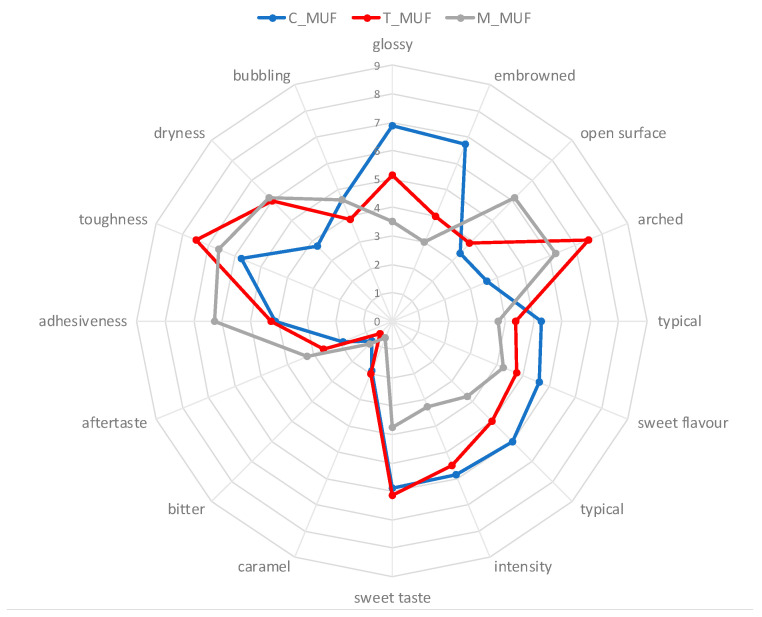
Sensory profile of C_MUF, M_MUF and T_MUF muffin samples. C_MUF = Muffin prepared by C_BP; M_MUF = Muffin prepared by M_BP; T_MUF = Muffin prepared by T_BP. 0-9 = Judge scores from 10-point structured scale.

Sensory profiles for C_MUF, T_MUF and M_MUF muffins are shown on Figure 9.

The main appearance descriptors were found to be the presence of a glossy crust, irregularities and openness in crust surface rate, and an arched shape of the muffin top surface. The main olfactory descriptor was muffin sweet flavor, while the principal taste descriptors were typical muffin taste, general taste, and sweet taste intensity; textural descriptors adhesiveness, toughness, mouthfeel dryness, and bubbling were all actively recognized by panelists. T_MUF and M_MUF muffins differentiated from control (C_MUF) for lower glossy crust and browning, while showing higher values of open surface and arched top surface. Flavor descriptors did not show significant differences, and taste profile showed generally lower values for T_MUF and M_MUF muffins as compared to the control. Textural descriptors were higher for modified muffins from control.

**Figure 10:**
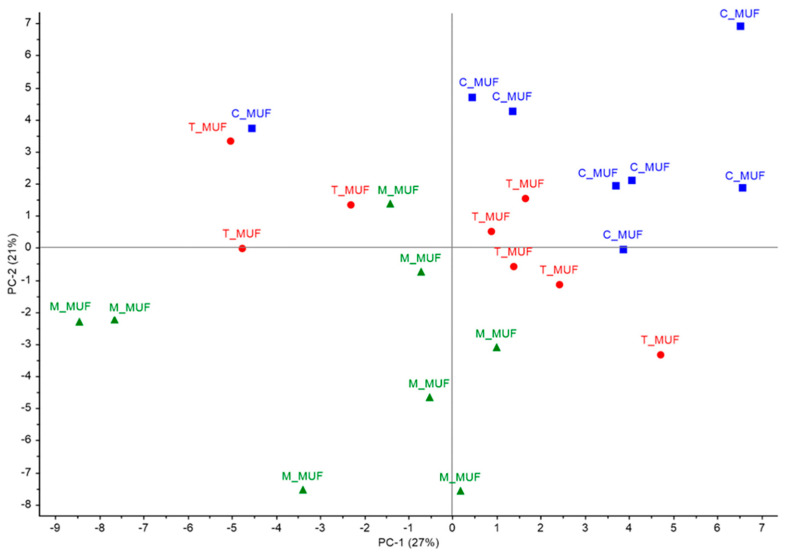
PCA Score plot of sensory evaluation of C_MUF, M_MUF and T_MUF muffin samples. C_MUF = Muffin prepared by C_BP; M_MUF = Muffin prepared by M_BP; T_MUF = Muffin prepared by T_BP.

**Figure 11:**
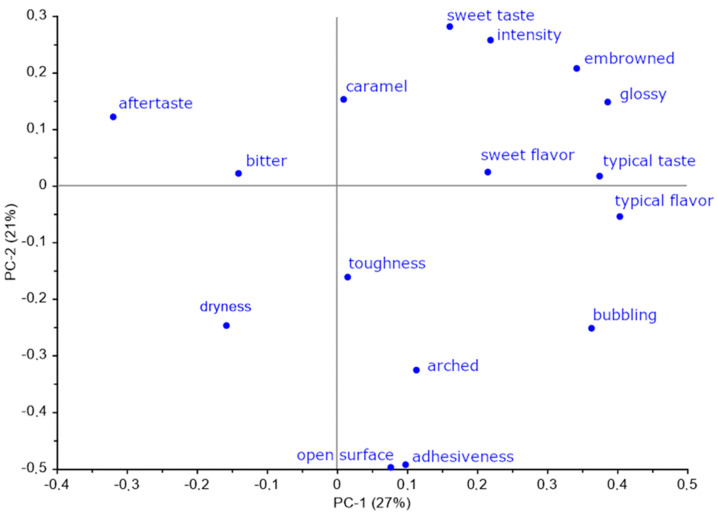
PCA loading plot of sensory attributes of muffin samples.

PCA (Principal Component Analysis) was performed on sensory data in order to explore variability between descriptors. Analysis was performed on all descriptors observed, with the first principal component explaining 27% of total variation and second component expressing 21% of total variation. PCA score results (Figure 10) show a good representation of muffin groups, with T_MUF and M_MUF types partially overlapping but quite clearly separated from control muffin group, that concentrates almost completely in the positive quadrant of both principal components. Such group is correlated (Figure 11) with presence of glossy and embrowned crust, sweet taste and flavor, typical taste and flavor, and general taste intensity. T_MUF muffins appear to be correlated to caramel taste intensity, sweet flavor, and bitter taste intensity. M_MUF muffins, on the contrary, appear more correlated the arched top surface descriptor, along with structural toughness and dryness and irregularities and openings in crust surface.

## 4. Conclusions

Multi-functional solid mixtures with antioxidant properties were synthesized, characterized, and proposed as innovative leavening agents for muffin production. The PXRD analysis demonstrated the formation of two multifunctional solid mixtures, CT_MA and CT_TA, consisting of a soluble polymorph of (+)-catechin (catechin tetrahydrate) and two organic acids (mucic and tartaric acid, respectively). The XRD pattern evaluation allowed us to suppose the maintenance of the individual properties of both components (i.e., baking and antioxidant activity). These aspects were confirmed by evaluating the antioxidant and rheological features of the leavening formulations. The commercial baking agent and its derived muffin samples showed negligible antioxidant properties and lower mechanical features in comparison to their innovative counterparts. In this sense, the replacement of phosphate additives with the new baking powder could improve the quality of the product either under the technological or under the nutritional point of view.

On the contrary, GC-MS, as well as taste analyses, revealed for modified muffins fewer volatile compounds and worse sensory traits, in comparison to the control; flavor descriptors did not show significant differences while textural descriptors were higher. Inferior taste characteristics surely represent a drawback, implying a lower consumer acceptance; however, it should be underlined that commercial formulations always include flavoring agents, (i.e., vanillin), while the CT_MA- and the CT_TA-based powders did not consider any flavor additive. It follows that with a more comprehensive formulation of the proposed leavening powders, the improvement of the taste features should be easily attainable.

Keeping in mind that, excluding infants, baked products are the main food categories contributing to the total P dietary exposure, the replacement of phosphate additives with a new baking ingredient could limit the overall intake. In this sense, it should be considered that the indication of the phosphorus concentration is not mandatory on the food labels as well as the use of other ingredients containing phosphorus. Only the presence of additives must be labelled, but not their quantities.

Moreover, the improved antioxidant and mechanical features of such innovative agents represent important advantages and the possibility to obtain the singular components from agro-food wastes, makes their exploitation even more attractive.

Further studies should be necessary to evaluate carefully all the ingredients included in the formulation, in order to correct specific sensory defects.

## Figures and Tables

**Table 1:** Muffin sensory descriptor list.

Category	Descriptor	Definition
**Appearance**	Glossy	Presence of glossy crust
	Embrowned	Presence and intensity of crust browning
	Open surface	Irregularities and openings in crust surface
	Arched	Shape of muffin top surface
**Olfactory**	Typical flavor	Product typical flavor intensity
	Sweet flavor	Sweetness intensity
**Taste**	Typical taste	Product typical taste intensity
	Intensity	General taste intensity
	Sweet taste	Sweet taste intensity
	Caramel	Caramel taste intensity
	Bitter	Bitter taste intensity
	Aftertaste	General aftertaste intensity
**Texture**	Adhesiveness	Adhesion rate to mouth during test
	Toughness	Chewing resistance rate
	Dryness	Dry mouthfeel impression
	Bubbling	Presence of empty areas inside product

**Table 2:** Antioxidant performances of baking powders. Data are presented asmean ± SD (*n* = 3), different letters in the same columns are statistically different (*p* < 0.05).

Baking	TPC (meq CT/g Baking)	TAC (meq CT/g Baking)	IC_50_ (mg mL^−1^)	
			DPPH Radical	ABTS Radical
**T_BP**	0.0298 ± 0.021 ^b^	0.0081 ± 0.0009 ^b^	2.358 ± 0.076 ^a^	0.149 ± 0.011 ^a^
**M_BP**	0.0530 ± 0.035 ^a^	0.0119 ± 0.0011 ^a^	0.518 ± 0.015 ^b^	0.112 ± 0.010 ^b^
**C_BP**	-	-	-	-
**Positive control** Ascorbic acid		0.1086 ± 0.0124	4.785 ± 0.126	1.654 ± 0.099

TPC = Total Phenolic Content; TAC = Total Antioxidant Capacity; CT = Catechin; DPPH = 2,2′-difenyl-1-picrylidrazil; ABTS = 2,2′-azino-bis (3-etylbenzotiazolin-6-sulphonic).M_BP = (+)-catechin_mucic acid-based baking powder; T_BP = (+)-catechin_tartaric acid-based baking powder; C_BP = commercial baking powder. (-) = not detected.

**Table 3:** Antioxidant properties of the prepared muffins. Data are presented as mean ± SD (*n* = 3), different letters in the same columns are statistically different (*p* < 0.05).

Muffin	TPC (MeqCT/g Muffin)	TAC (MeqCT/g Muffin)	IC_50_(mg mL^−1^)	
			DPPH Radical	ABTS Radical
**T_MUF**	0.00175 ± 0.00012 ^a^	0.00107 ± 0.00007 ^a^	1.236 ± 0.027 ^b^	0.098 ± 0.004 ^b^
**M_MUF**	0.00120 ± 0.00010 ^b^	0.00062 ± 0.00003 ^b^	2.251 ± 0.035 ^a^	0.789 ± 0.013 ^a^
**C_MUF**	-	-	-	-
**Positive control** Ascorbic acid		0.1086 ± 0.0124	4.785 ± 0.126	1.654 ± 0.099

TPC = Total Phenolic Content; TAC = Total Antioxidant Capacity; CT = Catechin; DPPH = 2,2′-difenyl-1- picrylidrazil; ABTS = 2,2′-azino-bis(3-etylbenzotiazolin-6-sulphonic).C_MUF = Muffin prepared by C_BP; M_MUF = Muffin prepared by M_BP; T_MUF = Muffin prepared by T_BP. (-) = not detected.

**Table 4:** Identified volatile compounds and their retention times in three different muffin formulations. Data represent mean ± SD (*n* = 3).

No	Analyte	Method of Identification	t_R_ (min)	M_MUF % *	T_MUF % *	C_MUF % *
**1**	1-hydroxy-2-propanone	α	3.25	-	-	15.29 ± 1.06
**2**	2-Methylbutanal	α, β	3.56	-	1.39 ± 0.25	-
**3**	3-Methylbutanal	α, β	3.72	2.39 ± 0.13	2.89 ± 0.08	1.92 ± 0.16
**4**	Pyrazine	α	4.56	0.62 ± 0.09	0.82 ± 0.12	0.43 ± 0.1
**5**	Pyridine	α, β	4.91	0.43 ± 0.06	0.18 ± 0.05	-
**6**	Toluene	α, β	5.6	1.53 ± 0.16	1.71 ± 0.11	0.88 ± 0.09
**7**	1-Pentanol	α	5.69	-	5.18 ± 0.15	1.41 ± 0.13
**8**	Hexanal	α, β	6.98	28.29 ± 0.55	29.67 ± 0.08	17.37 ± 0.56
**9**	Methylpyrazine	α	8.2	-	-	0.78 ± 0.15
**10**	2-Furancarboxaldehyde	α, β	9.07	-	-	0.32 ± 0.12
**11**	2-Furanmethanol	α, β	10.87	-	0.58 ± 0.26	13.66 ± 0.73
**12**	1,3-dimethylbenzene	α, β	11.34	3.45 ± 0.59	2.47 ± 0.31	-
**13**	2-Heptanone	α	13.04	4.79 ± 0.58	5.75 ± 0.25	2.84 ± 0.28
**14**	Heptanal	α, β	13.7	5.64 ± 0.25	4.21 ± 0.16	4.75 ± 0.22
**15**	2,5-dimethylpyrazine	α	14.15	13.17 ± 0.65	10.58 ± 0.17	4.22 ± 0.44
**16**	2(5H)-Furanone	α	14.58	-	5.33 ± 0.43	4.76 ± 0.1
**17**	Benzaldehyde	α, β	17.08	-	1.82 ± 0.41	0.52 ± 0.12
**18**	1-octen-3-ol	α, β	18.36	3.54 ± 0.06	2.61 ± 0.07	1.91 ± 0.17
**19**	2-pentylfuran	α	18.84	10.69 ± 0.48	8.72 ± 0.15	8.35 ± 0.29
**20**	Decane	α	19.26	3.24 ± 0.46	2.71 ± 0.12	2.22 ± 0.14
**21**	Octanal	α, β	19.45	-	-	3.61 ± 0.15
**22**	Benzene,1-methyl-4-(1-ethylethyl)	α	20.38	1.4 ± 0.14	1.22 ± 0.11	0.75 ± 0.11
**23**	2-Ethyl-1-hexanol	α	20.86	-	-	0.71 ± 0.21
**24**	2-Nonanone	α	23.59	1.81 ± 0.04	1.29 ± 0.06	1.09 ± 0.05
**25**	Undecane	α	23.88	5.98 ± 0.24	3.34 ± 0.13	3.15 ± 0.28
**26**	Nonanal	α, β	24.1	7.69 ± 0.14	4.21 ± 0.08	5.88 ± 0.16
**27**	Dodecane	α	27.89	3.16 ± 0.25	1.87 ± 0.11	1.58 ± 0.06
**28**	Tetradecane	α	34.87	-	-	0.65 ± 0.03
**29**	2,5-dibutylthiophene	α	39.74	1.9 ± 0.15	0.77 ± 0.02	0.40 ± 0.06

* relative peak area percentage. ^α^ Identification by NIST08 library. ^β^ Identification based on standard samples; C_MUF = Muffin prepared by C_BP; M_MUF = Muffin prepared by M_BP; T_MUF = Muffin prepared by T_BP. (-) = not detected.
